# Perceptions of women towards screening for intimate partner violence

**DOI:** 10.4102/phcfm.v14i1.3527

**Published:** 2022-09-14

**Authors:** Akintunde O. Akinyugha, Adebusola Akinyugha, Adesola O. Kareem, Abiodun J. Kareem, Modupe O. Ajewole, Elohor J. Orji, Adedeji Ogedengbe, Festus R. Babalola, Ruth O. Ayodele, Olawale J. Oladimeji

**Affiliations:** 1 Department of Family Medicine, Federal Medical Centre, Owo, Nigeria; 2 Registry Unit, Federal University of Technology, Akure, Nigeria; 3 Department of Community Medicine, Federal Medical Centre, Owo, Nigeria; 4 Department of Paediatrics, Federal Medical Centre, Owo, Nigeria; 5 Policy and Communication Unit, Academy for Health Development, Ile-Ife, Nigeria

**Keywords:** intimate partner violence, perception, prevalence, screening, women

## Abstract

**Background:**

Intimate partner violence (IPV) is an under-diagnosed public health problem affecting women with attendant negative bio-psycho-social ramifications, and unfortunately there is no universally agreed recommendation for routine hospital IPV screening currently.

**Aim:**

This study was carried out to determine the prevalence of IPV among women and their perceptions towards screening.

**Setting:**

The study was carried out in a hospital in Southwest, Nigeria.

**Methods:**

The study was a descriptive, cross-sectional study of 347 consenting women. Respondents were recruited using systematic random sampling. Data were collected using questionnaire adapted from the World Health Organization’s (WHO) Multi-Country Study Questionnaire on Women’s Health and Domestic Violence against women. Descriptive and inferential statistics were used and a *p*-value < 5% was considered significant.

**Results:**

The prevalence of IPV among the sample was 71.2%. The most common IPV pattern was controlling behaviour (49.6%) while sexual violence (19.6%) was the least. The majority (85.0%) of the respondents agreed that routine IPV screening should be done for women while 96.5% believed that it would enable doctors to help identify victims. The belief that it will help physicians in making a correct diagnosis, shared by 10.7% of the respondents, was statistically significant (odd ratio [OR] = 2.592, 95% confidence interval [CI] = 1.180–5.694, *p*-value = 0.018). A total of 37 respondents (10.7%) were about three times more likely to have experienced IPV than others.

**Conclusion:**

There was a high prevalence of IPV and the women are open to routine hospital IPV screening, with the belief that it will help physicians to make an accurate diagnosis of IPV.

**Contribution:**

This research was done by majority of family health specialists, in Nigeria, an African country. The focus of the research was distinctly with an African perspective, in the field of family medicine which has a public health implication and effect on the community.

## Introduction

Intimate partner violence (IPV) is one of the most common forms of violence against women and includes physical, emotional, sexual abuse and controlling behaviours by an intimate partner.^1^ It occurs all over the world with no socio-economic, religious or cultural barriers.^1^ An intimate partner is a person with whom one has a close personal relationship that can be characterised by emotional connectedness, regular contact, ongoing physical contact and/or sexual contact, identity as a couple, familiarity and knowledge about each other’s lives.^2^ Intimate partners include current or former spouses, boyfriends or girlfriends, dating partners or sexual partners who may or may not be living together.

In the past, IPV was considered mainly a social and minor issue affecting only relatively few women and not of importance to the health sector. However, becasue of the increasing awareness of its health consequences and its effects on the family, it has now been recognised to be of significant public health importance affecting every part of the society.^1,2,3,4,5^ Despite the health consequences of IPV, the response of the health sector in many countries, particularly the developing countries is inadequate.^6^

Despite the numerous studies done revealing the magnitude of IPV worldwide, there has been no consensus among professional health bodies on the need for screening all women presenting to healthcare facilities for IPV.^7^ For instance, the US Preventive Services Task Force (USPSTF) 2004 determined that evidence was insufficient to support screening women for IPV.^8^ However, in a review of new evidence on the effectiveness of screening and interventions for women in healthcare settings in 2012 by the USPSTF, findings suggest that screening could reduce IPV and improve health outcomes,^9^ therefore, recommending universal screening of women by healthcare providers. On the contrary, a 2014 Cochrane review concluded that there was insufficient evidence for universal screening in healthcare settings. Moreover, the review also noted that though screening resulted in a rise in case detection, their numbers were relatively low in comparison to the prevalence of IPV, and the review detected neither improved outcomes for women nor harm from screening.^10^ However, some studies suggest that women may not be averse to healthcare providers particularly the physicians asking them about possible IPV experience,^11,12^ hence, the need for further research into the perception of adult females towards screening. Many cases go unidentified simply because the attending physician failed to ask.^12^ Reasons for this include lack of training and feeling of inadequacy in handling cases of IPV, a wrong perception that it is only a social and not a health issue, thinking that affected women may not want to disclose their experiences to the doctor, fear of offending the patients, thinking that it is not part of a doctor’s duty to manage partners’ violence, the physician’s personal, religious and/or cultural beliefs and physicians’ apathy in general.^13^

This study, therefore, aimed to determine the perception of women towards screening for IPV, and the objectives are to determine the prevalence of IPV and the factors affecting the perception towards screening for IPV among women. The information from this study can be used in making policies that will help to reduce the burden and impact of IPV.

## Methods

### Study design

This was a hospital-based, descriptive, cross-sectional study.

### Setting

The research was carried out in Federal Medical Centre, Owo, Ondo State. The Federal Medical Centre Owo (FMC OWO) is a 280-bedded tertiary healthcare facility, which offers general and specialised care in various fields to patients from Ondo, Ekiti, Osun, Kogi and Edo States with the General Outpatient Clinic (GOPC) and the Emergency departments (ED) serving as the major points of contact and entry for diverse patients. The departments within the hospital are Family medicine, Emergency medical services, Internal medicine, Obstetrics and Gynaecology, Paediatrics, Community Health, Dental services, Pathology, Psychiatry, Radiology, Physiotherapy, Dietetics, Laboratory Services and Surgery and its subspecialties. The hospital offers residency training for doctors in Family medicine, Internal Medicine, General Surgery, Orthopaedics, Paediatrics and Obstetrics and Gynaecology. The hospital has a main laboratory and a references laboratory where several investigations are done daily including biochemical, microbiological, pathological, as well as a functional 24-h blood transfusion services.

The Family Medicine Department oversees the GOPC where patients are attended to by both the Consultant Family Physicians and the residents in training with a patient-centred care philosophy.

Owo is one of the local governments in Ondo State, Southwestern Nigeria. Owo local government area (LGA) lies within latitude 7.1833ºN and longitude 5.5833ºE, and it is about 50 km from Akure, the capital of Ondo State and 250 km from Lagos.^14^ Owo LGA occupies a land area of about 636 km² and is bounded by Emure-Ise-Orun LGA of Ekiti State to the North, Akure and Idanre LGAs of Ondo State to the East and South, respectively, while Ose LGA of Ondo State forms the border to the West and part of the South. According to the 2010 population census, the population of Owo is 261 131, and it was projected to be 300 000 in 2016.^15^ The people are predominantly of the Yoruba tribe with few Igbos, Ebiras and Igalas. The people work predominantly as civil servants, farmers, traders and artisans.

### Study population

The study participants were recruited from among registered female patients aged 18 years and above, attending the GOPC of Federal Medical Centre Owo.

### Sample size determination

The sample size was calculated using the Dobson formula^16^ drawing on a study by Nigeria Demographic and Health Survey 2013^17^, where the prevalence of IPV was 31.2%. A 5% margin of error, a confidence interval (CI) of 95%, a non-response rate of 5% and a minimum sample size of 347 participants were calculated:

$$n=\frac{Z_{a}^{2}(P)(1-P)}{d^{2}}$$ [Eqn 1]

### Sampling strategy

The inclusion criteria included women aged 18 years and above who gave consent and had or have had heterosexual intimate partners in the previous one year. Eligible women who had mental or severe medical illnesses that rendered them incapable of participating in the study were excluded. A total of 347 women were recruited for the study.

### Sampling technique

A systematic random sampling method was used in the selection of the study participants. An average of 100 patients attended the GOPC per day from the hospital records of which about 70 were adults. The female-to-male ratio from the records was approximately 2:1. Therefore, an average of 47 adult female patients is seen per day. The data collection was over eight weeks. All the 347 respondents were recruited in eight weeks, i.e. 347/8 = 43 respondents per week, hence nine patients per day. The sample interval was 5 (43/9 = 4.7). Each day, the first registered eligible consenting subject who fulfilled the inclusion criteria was recruited; this was followed by every fifth eligible consenting subject until the daily quota of nine was completed. For every eligible prospective respondent who declined to consent, the next eligible consenting woman was recruited in her stead. The study was conducted from 01 October 2017 until 30 November 2017.

### Data collection

Before the commencement of the study, the questionnaire was pretested among 34 women attending the GOPC of the State General hospital in the same locality as the study centre. The administration took between 10 and 15 min for each subject. The study was conducted using the pre-tested semi-structured, interviewer-administered questionnaire, which was also translated into the local language (Yoruba language) for those who did not understand the English language. The questionnaires were administered directly to eligible consenting subjects after written informed consent was obtained. The interview was conducted after consultation in a separate consulting room dedicated to the study for the sake of confidentiality. Each completed questionnaire was coded and kept in a well-secured bag and the data obtained were entered into the computer secured with a password. The questionnaires were administered directly by the principal investigator because of the sensitive nature of the subject studied. The data were cross-checked within 24 h for completeness.

The questionnaire was adapted from the questionnaire developed by the World Health Organization (WHO) in a Multi-Country Study on Women’s Health and Domestic Violence against Women, which is cross-culturally valid.^6^ The WHO Multi-Country study questionnaire was the outcome of a long process of discussion and consultation. Following an extensive review of a range of pre-existing study instruments and consultation with technical experts in specific areas (including violence against women, reproductive health, mental health, tobacco use and alcohol use), the core research team developed a first draft of the questionnaire. This was reviewed by the expert steering committee and experts in relevant fields, and suggestions for revision were incorporated. The revised questionnaire was then reviewed by the country teams during an international meeting where discussion focused on incorporating country priorities and achieving a balance between exhaustively exploring specific issues and compiling less detailed information on a range of issues. The questionnaire was then translated and pretested in six countries (Bangladesh, Brazil, Namibia, Samoa, Thailand and the United Republic of Tanzania). The experiences from these pre-tests were reviewed at the third meeting of the research teams where further revisions to the questionnaire were made. Following a final pre-test, the questionnaire for the study was completed as version 9.9 and was used in Bangladesh, Brazil, Ethiopia, Japan, Namibia, Peru, Samoa, Thailand and the United Republic of Tanzania. An updated version of the questionnaire (version 10), which incorporated the experience in the countries, was used in Serbia and Montenegro before the final approval.

The modifications made in the questionnaire for the purpose of this study included questions on the socio-demographic data, husband’s or partner’s attitude to IPV and subject’s financial autonomy. The questionnaire had three sections – A to C.

Section A was on the socio-demographic variables such as the subjects’ and their partners’ age, level of education, occupation and social class. The social class was assigned using the Ogunlesi socioeconomic classification.^18^ Social class was awarded based on the educational attainment and occupation of the partners. The mean of the four scores (two for the male and two for the female) to the nearest whole number was the social class assigned to the partners. Social classes I–II were considered high social class, social class III was middle class, while social classes IV–V were considered low social class.

Section B was on the experience of partner violence: this was categorised into four groupings – partner’s controlling behaviour, emotional violence, physical violence and sexual violence. Screening for partner’s controlling behaviour included asking whether a spouse or partner had ever kept her from seeing her friends, restricted her contact with her family, insisted on knowing where she is always, got angry if she speaks with other men, accused her of being unfaithful and whether he controls her access to healthcare? A ‘Yes’ response to one or more of these questions listed suggested the presence of the partner’s controlling behaviour.

Questions on emotional abuse included whether a spouse or partner had ever insulted her or made her feel bad about herself, if the latter had ever humiliated or belittled her in front of others, intimidated or scared her on purpose or had threatened to hurt her or hurt someone she cares about. A ‘Yes’ response to any of this indicated emotional abuse.

For physical violence, a respondent was asked whether a spouse or partner had ever slapped, kicked, dragged or beaten her, choked her on purpose, thrown something at her that could hurt her, threatened her with or use a dangerous weapon or object against her and if any of these had occurred in the past 12 months. A ‘Yes’ response to one or more of these questions indicated physical violence.

For sexual violence, a respondent was asked whether a spouse or a partner had ever physically forced her to have sexual intercourse against her will, whether she had sexual intercourse with the latter because she was afraid of what her partner might do and whether she had been forced to do something sexual she found degrading or humiliating. Also, if any of these had occurred in the past 12 months. A ‘Yes’ response indicated sexual violence. Questions on injuries sustained and the use of healthcare facilities because of IPV were also included in this section.

Section C was on perception towards screening for IPV. Respondents were asked if they have ever been asked by a doctor or other health workers about their experience of IPV during any prior hospital visit. Respondents were asked if physicians and health workers should routinely ask adult female patients about IPV for which the Likert scale was scored 1–5; strongly agree was scored 1 point; agree had a score of 2 points; undecided had 3 points; disagree had 4 points and strongly disagree had 5 points. They were also asked to give the reason(s) for their answer.

### Data analysis

The data obtained were analysed using the Statistical Package for Social Sciences (SPSS) for Windows software version 22 (SPSS 22, Chicago).^19^ Descriptive data were presented using tables and charts. The prevalence of IPV experience was summarised using proportions. Associations between the categorical independent variables and perception towards screening for IPV were assessed with the chi-square test. Multivariate regression analysis was done to identify independent predictors of perception towards screening for IPV. The level of significance for all the tests was 5% (95% CI).

### Ethical considerations

Ethical clearance was obtained from the Ethics and Research Committee of Federal Medical Centre, Owo, Ondo State, with reference number FMC/OW/380/VOL.XLVII/122. Written informed consent was obtained from all the respondents after explaining the nature of the study to them. Confidentiality of all the divulged information was assured by maintaining anonymity on the questionnaires and ensuring that only the researcher had access to the respondents’ information. Each participant was informed that she could terminate the interview and pull out of the research at any stage without any consequences.

## Results

A total of 347 women were recruited for the study. The respondents’ mean (± standard deviation [s.d.]) age was 41.8 (± 15.6) years, with age group < 30 years being the largest proportion, 111 (32.0%), while the age group 50–59 years had the lowest proportion 49 (14.1%). More than half (54.2%) of the respondents were married, while only 2.3% of the respondents were cohabiting. Among the ever-married participants, about two-thirds were monogamous. More than three-quarters (81.0%) of the respondents were from the Yoruba tribe, while Christianity (91.4%) was the dominant religion. The middle class constituted the highest proportion (37.5%) of the respondents. The majority (60.5%) earned above the national minimum wage (Table 1).

**TABLE 1:** Socio-demographic characteristics of the respondents.

Variables	Frequency (*n* = 347)	%
**Age in years**		
< 30	111	32.0
30–39	59	17.0
40–49	66	19.0
50–59	49	14.1
> 60	62	17.9
**Marital status**		
Single	69	19.9
Married	188	54.2
Separated or divorced	24	6.9
Widowed	58	16.7
Cohabiting	8	2.3
**Tribe**		
Yoruba	281	81.0
Hausa	2	0.6
Igbo	22	6.3
Other†	42	12.1
**Religion**		
Christianity	317	91.4
Islamic	30	8.6
**Socio-economic status**		
High class	99	28.5
Middle class	130	37.5
Low class	118	34.0
**Average monthly income (₦)**		
≤ 18.000	137	39.5
≥ 18.000	210	60.5

₦, Nigerian Naira

† , Ebira, Igala, Edo, Ijaw.

The prevalence of IPV experienced among the respondents was 71.2%, while 28.8% had never experienced any form of IPV in the previous 12 months. Table 2 showed the types of IPV experienced by the respondents. Controlling behaviour (49.6%) was the commonest type of IPV experienced, while sexual violence (19.6%) was the least.

**TABLE 2:** Types of intimate partner violence experienced by respondents.

Variables	Frequency (*n* = 347)	%
**Controlling behaviour IPV**		
Yes	172	49.6
No	175	50.4
**Psychological IPV**		
Yes	163	47.0
No	184	53.0
**Physical IPV**		
Yes	114	32.9
No	233	67.1
**Sexual IPV**		
Yes	68	19.6
No	279	80.4

IPV, Intimate partner violence

The majority of respondents 309 (89.0%) had never been screened for IPV in the previous 12 months, while only 38 (11.0%) of the respondents had ever been asked by a doctor or other health workers about their experience of IPV during any prior hospital visit in the previous 12 months.

**FIGURE 1:**
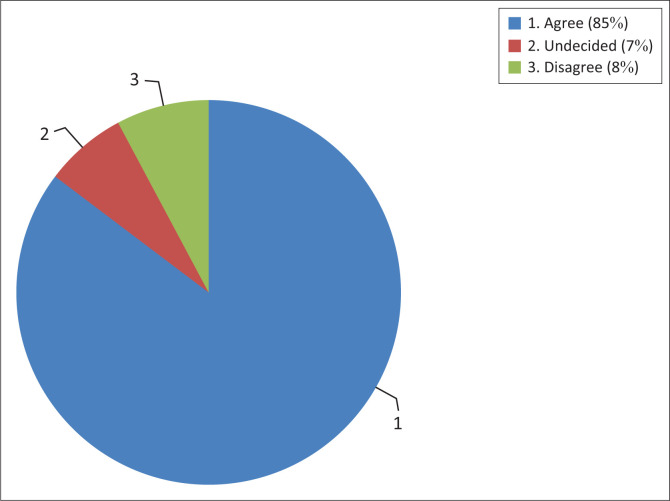
Perception of respondents towards routine intimate partner violence screening.

The perception of the respondents towards routine IPV screening for every adult female that presented to the GOC is shown in Figure 1. An overwhelming majority of the respondents 296 (85.0%) agreed that all women should be routinely screened for IPV, while 27 (8.0%) respondents disagreed with routine screening.

**FIGURE 2:**
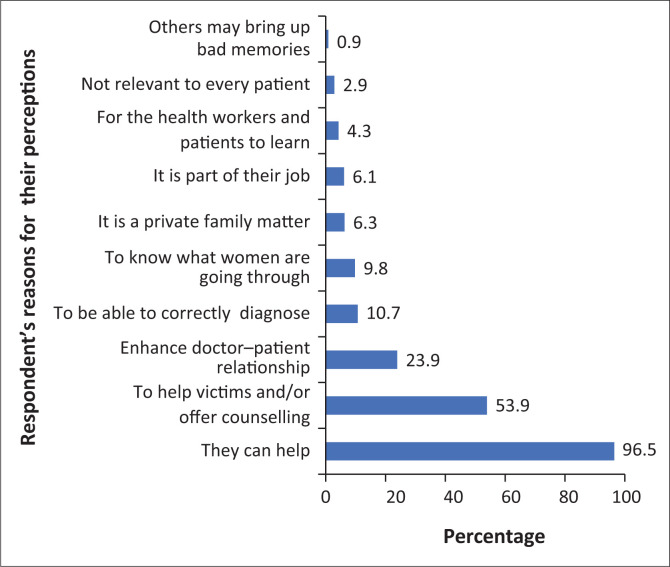
Respondents’ reasons for their perception on routine intimate partner violence screening.

The reasons for the respondents’ positive perception towards routine IPV screening are shown in Figure 2. Most of the respondents 335 (96.5%) believed that routine IPV screening would enable doctors to help the identified victims. More than half 187 (53.9%) of them felt that it would enable doctors to offer the IPV victims appropriate counselling, while 21 (6.1%) respondents felt that routine IPV screening should be part of the job or responsibilities of doctors by default, and 37 (10.7%) felt it would assist doctors in making the right diagnosis. Conversely, 22 (6.3%) respondents believed it was a private family matter while 3 (0.9%) respondents believed it could bring up bad memories in the patients’ minds. It should be noted, however, that many of the respondents gave multiple responses.

Table 3 showed the relationship between IPV and the perception towards IPV screening among the respondents. The only statistically significant finding was among respondents who thought that routine IPV screening will help doctors arrive at the right diagnosis (odds ratio [OR] = 2.592, CI = 1.180–5.694, *p*-value = 0.018). These respondents were about three times more likely to have IPV screening than other respondents.

**TABLE 3:** Relationship of intimate partner violence and perception towards screening among respondents.

Variables	s.e.	df	*χ* ^2^	*P* value	Odd ratio	95% confidence interval	
**To offer counselling**							
Yes	0.337	1	2.166	0.141	1.643	0.848	3.181
No	-	-	-	-	1	-	-
**It is part of their job**							
Yes	0.598	1	0.383	0.536	0.691	0.214	2.229
No	-	-	-	-	1	-	-
**It enhances doctor–patient relationship**							
Yes	0.335	1	0.009	0.925	0.969	0.502	1.869
No	-	-	-	-	1	-	-
**To be able to correctly diagnose**							
Yes	0.401	1	5.632	0.018	2.592	1.180	5.694
No	-	-	-	-	1	-	-
**To know what women are going through**							
Yes	0.475	1	0.268	0.605	0.782	0.308	1.983
No	-	-	-	-	1	-	-
**For the health workers and patients to learn**							
Yes	0.686	1	0.002	0.963	0.968	0.252	3.718
No	-	-	-	-	1	-	-
**It is a private matter**							
Yes	0.952	1	2.198	0.138	4.102	0.635	26.506
No	-	-	-	-	1	-	-
**They can help**							
Yes	1.017	1	0.760	0.383	0.412	0.056	3.026
No	-	-	-	-	1	-	-
**Not relevant to every patient**							
Yes	1.042	1	0.835	0.361	0.386	0.050	2.976
No	-	-	-	-	1	-	-
**May bring up bad memories**							
Yes	2318.3	1	0.000	0.999	46602.1	0.000	-
No	-	-	-	-	-	-	-
**Ever been asked by health workers about IPV**							
Yes	0.453	1	2.154	0.142	0.514	0.212	1.250
No	-	-	-	-	1	-	-

s.e., standard error; df, degrees of freedom; *χ*^2^, chi-square; IPV, intimate partner violence.

## Discussion

The mean (± SD) age of the respondents was 41.8 (± 15.6) years, which mirrors the Nigerian population pyramid as reported by the Nigerian Demographic and Health Survey (NDHS) carried out in 2013, which indicated that Nigeria comprises a relatively young population.^17^ Married women were the major participants in this study, and this is not surprising because most of the women were within the period of life when most women marry.^6^ The majority of the respondents belonged to the middle and low socio-economic class, which is a reflection of the socio-economic realities of the time.^17^ It might also be because most people in this socio-economic group patronise government-owned hospitals more than private hospitals as services rendered by the former are usually more affordable for them when compared to the latter.^20^

The prevalence of IPV in the previous 12 months found in this study was 71.2%, which was similar to a study in South East Nigeria.^21^ The prevalence in this study was higher than the reported prevalence in the developed parts of the world such as the Western Europe (5.0%), Southern Europe (4.0%), Central Asia (9.0%) and the North America (6.0%).^22^ This might be because of the higher level of awareness, enlightenment and criminalisation of acts constituting IPV in these regions.^23^ It was also higher than the study done among pregnant women in Egypt with a prevalence of 44.1%.^24^ The prevalence of IPV in this study was also higher than what was found in another study in Nigeria, with a prevalence of 46.0%, but the study was limited to physical IPV.^25^ The prevalence in this study was, however, lower than the prevalence among rural (97.0%) and urban (81.0%) communities in South East, Nigeria.^3^ This study^3^ was a community-based study with a larger sample size.

The trend of IPV pattern in this study was similar to that reported in the WHO Multi-Country study where the proportion of study participants who had experienced controlling behaviour (21% – 90%), psychological and/or emotional violence (20% – 75%), physical violence (13% – 61%) and sexual violence (6% – 59%)^6^ was in concordance to this study. Controlling behaviour was the predominant type of IPV found in this study. This could be because of male control over their spouses’ or partners’ behaviour as normative to a large extent in the study environment as in many other parts of the world.^6^ In most relationships in our setting, the male partner is usually older and therefore tends to want to control the female and the relationship dynamics. This was further supported by a study in Spain, which showed that controlling behaviour was more frequently reported among couples where the man was older than the woman.^26^ It was also reported that controlling behaviour could be culturally acceptable.^21^ Moreover, controlling behaviour is closely associated with other forms of violence.^6,26^ Contrary to the finding in this study, other studies reported psychological violence as more predominant.^3,27,28,29,30^ The difference in the studies was because the present study included controlling behaviour as a type of IPV, while the other studies focused on the other three types of IPV (psychological, physical and sexual violence).

Sexual violence was the least common pattern of IPV among the respondents in this study (19.6%). This finding was similar to several other studies.^6,21,25,31,32^ This could be because sexual issues are intimate matters and many of the participants are unwilling to divulge information about it. This prevalence was higher than that reported by NDHS (7.0%),^17^ WHO (13.0%)^33^ and a study in Nigeria with a sexual violence prevalence of 6.6%, 3.7% and 10.7%, respectively, in the previous 12 months.^32,34,35^ The difference could be because the present study was hospital based, while the other studies were community based. The sexual violence prevalence in this study was lower than the findings in Guinea (68.1%).^27^ The latter study was carried out among female clients in a family planning clinic and the higher prevalence of sexual violence might be because the women who were seeking family planning services were at high risk of IPV *ab initio.*^27^

From this study, it was found that 309 (89.0%) respondents had never been asked by any doctor or other healthcare practitioners about IPV experience in the previous 12 months during any hospital visits. This is similar to other studies that showed that few women reported being routinely asked about IPV by the physicians in the clinics or in the emergency room; hence many cases go undetected.^1,11,12^ Most of those that were previously screened were asked because they presented to the hospital with injuries sustained from trauma following physical IPV.^36^

An overwhelming majority of the respondents 296 (85.0%) agreed that all women should be routinely screened for IPV while less than one-tenth of the respondents 27 (8.0%) disagreed, and 24 (7.0%) were undecided. This finding suggested that women may not be opposed to being routinely questioned about their IPV experience. This was similar to a previous study that reported that women would welcome being screened by physicians in a confidential and supportive manner.^11^ The USPSTF cautions on routine screening for or against IPV.^8^ This was because there is insufficient evidence for screening, and for effective IPV screening, the screening tool must include sound psychometric properties.^8,37^ Another study reported that despite available screening tools identifying women experiencing IPV, there was no reduction in IPV or improvement in quality of life over 3–18 months,^38^ which, therefore, questioned the need for IPV screening. Despite the several challenges to IPV screening, many of these challenges can be addressed through the development of a systematic screening tool, proper training of health professionals, home visits and behavioural counselling interventions that address the risk factors for IPV.^37,38^

In developing a clinical and policy guideline as a response to IPV, the WHO found that respondents identified healthcare providers as the professionals they would most trust with disclosure of IPV.^39^ The most common reasons cited by the respondents who agreed were that routine IPV screening would enable physicians in identifying and helping the affected victims (96.5%), it would enable physicians to offer them necessary counsel (53.9%), and it would enhance patient–physician relationship (23.7%). All these are reasons given from previous studies that had recommended routine IPV screening in health care settings.^9,11,40,41^ However, another study had shown a contrary view that some women have personal discomfort with the topic, some physicians do not have the time, training, or privacy to screen, the fear of offending the patients and that IPV screening is not a clinician’s duty.^37^

The predictor for positive perception towards screening for IPV in this study was that the screening will help physicians in making the correct diagnosis. Of these respondents, 37 (10.7%) were three times more likely to allow IPV screening than others.

The present study showed that respondents disagreed with routine IPV screening (8.0%) or were undecided about IPV screening (7.0%) because it was not relevant to every patient (6.9%), it was a private family matter and therefore beyond the purview of physicians (6.3%) and that it could evoke bad memories in affected women (0.9%), which they would rather not want to talk about. The last two reasons have been proposed to be part of the factors responsible for recall biases in studies and therefore the underestimation of IPV prevalence^6,17,42^

### Limitations of the study

The limitation of this study is the effects of recall and reporting bias on the part of the respondents. Nevertheless, a high level of confidentiality and privacy was ensured to minimise the effect of the reporting bias while administering the study instrument. Also, the findings of this study might not be generalisable to the general populace because it is a hospital-based study. The strength of the study is the instrument used for investigating IPV, which was adapted from the Women’s Health and Life Experiences Questionnaire, a validated tool developed by the WHO for IPV research.

## Conclusion

In conclusion, IPV was highly prevalent among women presenting to hospital in Owo, Ondo State, and controlling behaviour was the most common form. The majority of the women would want routine IPV screening during hospital visits as it would help the physician in making the correct diagnosis. It is, therefore, recommended that routine IPV screening be incorporated into routine history taking among adult females in GOPCs. Likewise, a community-based study should be conducted to know the prevalence of IPV and perceptions of women towards IPV.
